# Building a Genetic Manipulation Tool Box for Orchid Biology: Identification of Constitutive Promoters and Application of CRISPR/Cas9 in the Orchid, *Dendrobium officinale*

**DOI:** 10.3389/fpls.2016.02036

**Published:** 2017-01-12

**Authors:** Ling Kui, Haitao Chen, Weixiong Zhang, Simei He, Zijun Xiong, Yesheng Zhang, Liang Yan, Chaofang Zhong, Fengmei He, Junwen Chen, Peng Zeng, Guanghui Zhang, Shengchao Yang, Yang Dong, Wen Wang, Jing Cai

**Affiliations:** ^1^State Key Laboratory of Genetic Resources and Evolution, Kunming Institute of Zoology, Chinese Academy of SciencesKunming, China; ^2^Nowbio Biotech Inc., KunmingChina; ^3^State Key Laboratory of Quality Research in Chinese Medicine, Institute of Chinese Medical Sciences, University of MacauMacau, China; ^4^National and Local Joint Engineering Research Center on Gemplasm Utilization and Innovation of Chinese Medicinal Materials in Southwest China, Yunnan Agricultural UniversityKunming, China; ^5^China National GeneBank, BGI-ShenzhenShenzhen, China; ^6^Pu’er Institute of Pu-er TeaPu’er, China; ^7^Key Laboratory of Molecular Biophysics of the Ministry of Education, College of Life Science and Technology, Huazhong University of Science and TechnologyWuhan, China; ^8^College of Horticulture and Landscape, Yunnan Agricultural UniversityKunming, China; ^9^Faculty of Life Science and Technology, Kunming University of Science and TechnologyKunming, China; ^10^Province Key Laboratory, Biological Big Data College, Yunnan Agricultural UniversityKunming, China; ^11^Shenzhen Key Laboratory for Orchid Conservation and Utilization, National Orchid Conservation Center of China and Orchid Conservation and Research Center of ShenzhenShenzhen, China

**Keywords:** *Dendrobium officinale*, transgene, vector construction, CRISPR/Cas9 gene editing, overexpression, lignocellulose biosynthesis, gene promoters

## Abstract

Orchidaceae is the second largest family of flowering plants, which is highly valued for its ornamental purposes and medicinal uses. *Dendrobium officinale* is a special orchid species that can grow without seed vernalization. Because the whole-genome sequence of *D. officinale* is publicly available, this species is poised to become a convenient research model for the evolutionary, developmental, and genetic studies of Orchidaceae. Despite these advantages, the methods of genetic manipulation are poorly developed in *D. officinale*. In this study, based on the previously developed *Agrobacterium*-mediated gene transformation system, we identified several highly efficient promoters for exogenous gene expression and successfully applied the CRISPR/Cas9 system for editing endogenous genes in the genome of *D. officinale*. These two basic techniques contribute to the genetic manipulation toolbox of Orchidaceae. The pCambia-1301-35SN vector containing the CaMV 35S promoter and the *β-glucuronidase* (*GUS*) and Superfolder green fluorescence protein (SG) as reporter genes were introduced into the plant tissues by the *Agrobacterium*-mediated transformation system. Fluorescence emission from the transformed plants confirmed the successful transcription and translation of SG genes into functional proteins. We compared the GUS activity under different promoters including four commonly used promoters (MtHP, CVMV, MMV and PCISV) with CaMV 35S promoter and found that MMV, CVMV, and PCISV were as effective as the 35S promoter. Furthermore, we applied the CRISPR/Cas9-mediated genome editing system successfully in *D. officinale*. By selecting five target genes (*C3H, C4H, 4CL, CCR, and IRX*) in the lignocellulose biosynthesis pathway, we showed that, for a given target, this system can generate edits (insertions, deletions, or substitutions) at a rate of 10 to 100%. These results showed that our two genetic manipulation tools can efficiently express exogenous genes and edit endogenous genes in *D. officinale*. These efficient research tools will not only help create novel *D. officinale* varieties, but will also facilitate the molecular genetic investigation of orchid biology.

## Introduction

The orchid family (Orchidaceae) is the second largest family of flowering plants with blooms that are often colorful and fragrant. This family also encompasses about 6–11% of all seed plants (Pillon and Chase, 2007). It is comprised of five subfamilies, 880 genera and more than 25,000 species worldwide (Stevens, 2001; Hsiao et al., 2011). Many wild and cultivated Orchidaceae flowers have been utilized in perfumery, horticulture, food, and traditional medicine (Gutierrez, 2010). There is thus interest in employing genetic manipulation to produce new orchid cultivars that have better resistance to pests, novel flower colors, higher productivity, larger inflorescence and longer post-harvest shelf-life.

There are multiple ways to manipulate plant gene expression using transgenic technology. We focused on two: overexpression of exogenous genes in the host and silencing of endogenous genes of the host. To achieve overexpression, many reports have published methods for introducing exogenous genes into various Orchidaceae species by bombardment or *Agrobacterium*-mediated transformation (Yu et al., 2001; Mishiba et al., 2005; Lu et al., 2007; Zhang et al., 2010; da Silva et al., 2011, 2016; Hsing et al., 2016; Phlaetita et al., 2015). To achieve gene silencing, just a few reports showed RNAi-mediated gene knock-down in *Dendrobium* Sonia (Liu et al., 2014) and *Oncidium* hybrid orchid (Liu et al., 2014). And it was reported that this RNAi-mediated approach had several limitations such as incomplete loss-of-function and extensive off-target activities in many plant species although the off-target activities has not been examined systematically in orchids (Xu et al., 2006). In comparison, gene silencing has also been achieved by gene editing using the new technology called CRISPR/Cas9 (clustered regularly interspaced short palindromic repeat/CRISPR-associated protein 9). This system has two major advantages: firstly, complete knock out of target gene instead of partial knock down due to dosage difference in RNAi (Barrangou et al., 2015); secondly, stable genomic change that can be easily maintained in the offspring which is an important advantage for application in breeding programs (Liu et al., 2016). Generally speaking, genome editing by CRISPR/Cas9 is achieved by firstly cutting the doubles strand DNA at target sites followed by repairing the double-stranded break (DSB) through non-homologous end-joining (NHEJ) or homology-directed repair (HDR) mechanisms (Shalem et al., 2015). In most applications, random insertion or deletion (indels) introduced by NHEJ causes will disrupt the open reading frame of protein-coding sequences or loss of the cis-regulatory elements (such as promoters or enhancers) in non-coding sequences (Liu et al., 2016). CRISPR/Cas9 has proven to be a powerful loss-of-function tool in many plants, such as rice, wheat, tobacco, maize, and *Arabidopsis* (Feng et al., 2013; Jiang et al., 2013; Nekrasov et al., 2013; Belhaj et al., 2015). Until now, no CRISPR/Cas9 system application has been reported in orchid plants. The application of CRISPR/Cas9-mediated genome editing in orchids may provide us more choices in orchid genetic improvement. For CRISPR/Cas9, the available genome sequence of host species could help select target sites with low off-target efficiency and increase the efficiency and accuracy of genome editing. Currently, there are only published genome sequences of two orchid species: *Dendrobium officinale* (Yan et al., 2015; Zhang G.Q. et al., 2016) and *Phalaenopsis equestris* (Cai et al., 2015).

*Dendrobium officinale (D. officinale)* is a top-ranked Chinese medicinal herb along with *Ganoderma, Ginseng* and *Cordyceps sinensis*. *D. officinale* has multiple pharmacological activities, such as immunomodulation (Gao et al., 2002), anti-tumor (Jin et al., 2010), anti-oxi (Jin et al., 2010), anti-oxidation (Yang et al., 2009), anti-fatigue (Lu et al., 2010), and protective effects on the renal function of experimental diabetic rats (Li et al., 2012). Compared with other Orchidaceae species, the sequenced and annotated genome of *D. officinale* (Yan et al., 2015; Zhang G.Q. et al., 2016), and the well-established transformation systems of the *Dendrobioum* genus (Teixeira da Silva et al., 2016) make it an ideal genetic manipulation model for the Orchidaceae family.

In this study, we identified several efficient promoters for over-expressing exogenous genes, and applied CRISPR/Cas9 mediated genome editing systems in *D. officinale*, based on an *Agrobacterium-*mediated transformation system of *Dendrobium*.

## Materials and Methods

### Overexpression Vectors Construction

**FIGURE 1:**
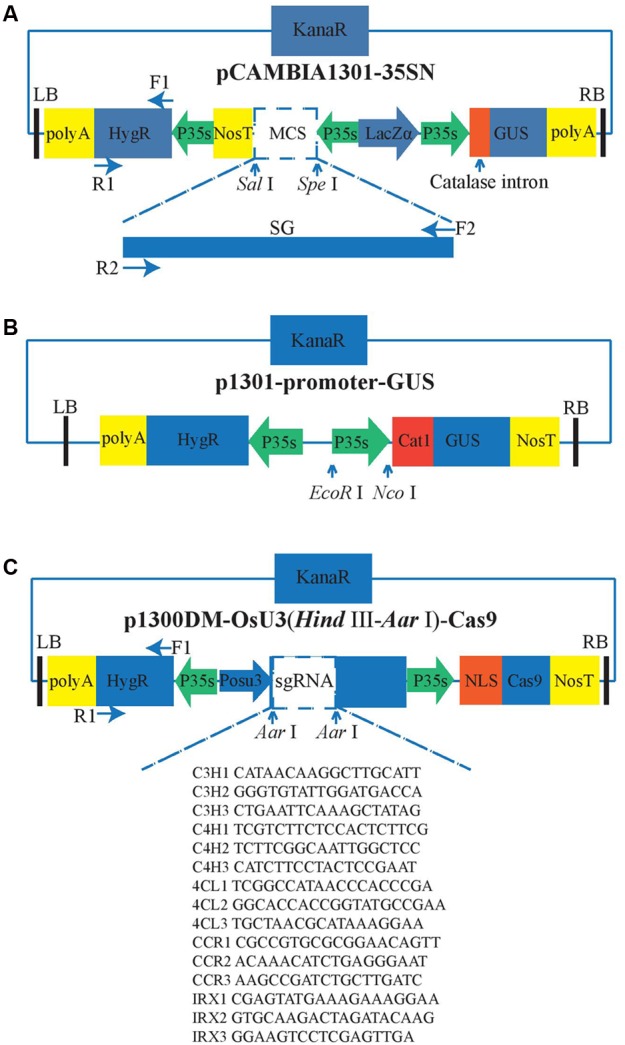
**Vector structure. The green arrows represent the promoters and the yellow rectangles represent the terminators, the blue rectangles represent the antibiotic selection markers, the black bars represent the border sequence of the Ti-plasmid. P35s means the CaMV 35S promoter; POsU3 means the OsU3 promoter. F and R are the primers used to amply the corresponding fragments. The arrows indicate the relative restriction sites that can be used for the insertion of the targets. (A)** Overexpression, **(B)** Five constitutively active promoters. The promoters which have been tested were inserted between EcoR I and Nco I. **(C)** Gene knock-out.

The vector pCambia1301-35SN is used for overexpression and can be classified as an *A. tumifaciens* Ti-plasmid vector because of the existence of the left border (LB) and right border (RB) (**Figure 1A**). The Ti-DNA region of this vector harbors *hygromycin phosphotransferase* (*HygR*) and *LacZα* as the selection marker genes and *β-glucuronidase* (*GUS*) as the reporter gene, which is interrupted by a *catalase intron.* The transcription of *GUS* gene and *HygR* gene are both driven by the CaMV 35S (35S) promoter and ended by the 35S’ terminator. The exogenous genes are integrated into the multiple cloning sites (MCS) at the restriction enzymes *Sal*I and *Spe*I recognition sites. The 35S promoter and *nitric oxide synthase* terminator (*Nos3′*) are used to control the open reading frame (ORF) of the exogenous gene. In our experiment, the DNA fragments containing the *SG* gene and *SpeI* and *SarI* restriction sites were cut by the restriction enzymes *SpeI* and *SarI*. The purified *SG* fragments were inserted into the linearized pCambia1301-35SN with the T4 DNA ligase (NEB, Kunming, China).

We attempted to identify four efficient promoters for gene overexpression as follows: The *Medicago truncatula* hypothetical protein (MtHP) promoter (Xiao et al., 2005), a promoter isolated from the cassava vein mosaic virus (CVMV) (Verdaguer et al., 1996), a promoter isolated from mirabilis mosaic virus (MMV) (Dey and Maiti, 1999), and the peanut chlorotic streak caulimovirus (PCISV) promoter (Maiti and Shepherd, 1998). These were cloned into the pCambia1301 vector including *GUS* marker gene (**Figure 1B**), and then infected the protocorms by *Agrobacterium*-mediated transformation. Finally, we compared the activity of *GUS* driven by different promoters with 35S promoter to find out which one can work efficiently in the transgenic system of *D. officinale*.

### CRISPR/Cas9 Construct Design and Targets Selection

To create a *D. officinale* CRISPR/Cas9 genome editing system, an RNA-guided genome editing vector line p1300DM-OsU3 (*Hin*d III-*Aar*I)-Cas9 (**Figure 1C**) was used for expressing engineered *sgRNA* and *Cas9* in *D. officinale* cells. This vector also belongs to the pCambia vector family. In this vector every element is essential except the extra spacer between two Aar1 restriction sites in sgRNA expression cassettes which are used for the integration of sgRNA fragments. As shown in **Figure 1C**, the sgRNA expression cassette is driven by the OsU3 promoter. This promoter is a small nuclear RNA (snRNA) promoter that initiates transcription at an adenine nucleotide and drives the high expression of sgRNAs (Shan et al., 2013; Ma et al., 2015). The Cas9, which is modified with plant-optimized codons (Cong et al., 2013; Mao et al., 2013), has an SV40 nuclear localization signal (*SV40 NLS*) (Brandén et al., 1999) that can guide the mature CRISPR/Cas9 complex into the cell nucleus. It is driven by the 35S promoter. Another copy of 35S promoter also drives the expression of *HygR*, and our lab’s previous experience of successful application of the same CRISPR/Cas9 system in rice has excluded the concern that using two 35S promoters for both Cas9 and HygR expression might result in gene silencing.

The abundance of lignocellulose is a key factor affecting the taste and popularity of *D. officinale* in the natural health product market. So we were interested in finding out whether we could reduce the content of lignocellulose by using the CRISPR/Cas9 system to knock out genes in the lignocellulose biosynthesis pathway. Based on the public genome sequence of *D. officinale* (Yan et al., 2015) and knowledge of lignocellulose biosynthesis (Bonawitz and Chapple, 2010), five genes (**Table 1**), *COUMARATE 3-HYDROXYLASE* (*C3H*) (Schoch et al., 2001; Ralph et al., 2006), *CINNAMATE4-HYDROXYLASE* (*C4H*) (Sewalt et al., 1997; Bonawitz and Chapple, 2010), *4-COUMARATE:COENZYME A LIGASE* (*4CL*) (Lee et al., 1997), *CINNAMOYL COENZYME A REDUCTASE* (*CCR*) (Goujon et al., 2003; Jackson et al., 2008; Bonawitz and Chapple, 2010) and *IRREGULAR XYLEM5* (*IRX*) (Taylor et al., 2003), participating in the lignocellulose biosynthesis process, were selected as target genes to explore whether our CRISPR/Cas9 system could work in *D. officinale* efficiently. We first obtained the sequences of five genes in *Arabidopsis thaliana* from the NCBI database and then found their orthologous genes by BLAST against the V2 genome assembly of *D. officinale* as reported by (Yan et al., 2015).

**Table 1:** The sgRNA oligos of target genes.

Gene	Full gene name	Oligos
*C3H*	Coumarate 3-hydroxylase	1. GGCACATAACAAGGCTTGCATTAAACAATGCAAGCCTTGTTATGT 2. GGCAGGGTGTATTGGATGACCAAAACTGGTCATCCAATACACCCT 3. GGCACTGAATTCAAAGCTATAGAAACCTATAGCTTTGAATTCAGT

*C4H*	Cinnamate 4-hydroxylase	1. GGCATCGTCTTCTCCACTCTTCGAAACCGAAGAGTGGAGAAGACGAT 2. GGCATCTTCGGCAATTGGCTCCAAACGGAGCCAATTGCCGAAGAT 3. GGCACATCTTCCTACTCCGAATAAACATTCGGAGTAGGAAGATGT

*4CL*	4-coumarate: coenzyme A ligase	1. GGCATCGGCCATAACCCACCCGAAAACTCGGGTGGGTTATGGCCGAT 2. GGCAGGCACCACCGGTATGCCGAAAAACTTCGGCATACCGGTGGTGCCT 3. GGCATGCTAACGCATAAAGGAAAAACTTCCTTTATGCGTTAGCAT

*CCR*	Cinnamoyl coenzyme A reductase	1. GGCACGCCGTGCGCGGAACAGTTAAACAACTGTTCCGCGCACGGCGT 2. GGCAACAAACATCTGAGGGAATAAACATTCCCTCAGATGTTTGTT 3. GGCAAAGCCGATCTGCTTGATCAAACGATCAAGCAGATCGGCTTT

*IRX*	Irregular xylem5	1. GGCACGAGTATGAAAGAAAGGAAAAACTTCCTTTCTTTCATACTCGT 2. GGCAGTGCAAGACTAGATACAAGAAACCTTGTATCTAGTCTTGCACT 3. GGCAGGAAGTCCTCGAGTTGAAAACTCAACTCGAGGACTTCCT

Three pairs of oligonucleotides targeted to different sites were designed for each gene to increase the possibility that the right gene was targeted (**Table 1**). The DNA sequences with the form AN18-20NGG were selected as the candidates and the NGG means the protospacer adjacent motifs (PAM). The specificity of these candidates are examined by BLAST search against the genome sequence of *D. Officinale.* In order to make the CRISPR/Cas9 constructs, two DNA oligos were chemically synthesized for each gRNA. The two oligos were complementary for the sequence corresponding to the spacer, and the sequences GGC and AAAC were added to the 5′ end of the forward and reverse oligos, respectively, to allow for the formation of cohesive ends of *Aar*I restriction sites following annealing. (See **Table 1** for oligo sequences). These coupled oligos were ligated into the vector p1300DM-OsU3 *(Hin*d III-*Aar*I), linearized by *Aar* I restriction enzyme digestion.

### *E. coli* and *A. tumefaciens* Transformation

The constructed vectors for overexpression, promoters test and genome editing were first amplified by transferring into *E. coli* competent cells (DH5α), incubated in the LB medium, which use *kanamycin* as a selection reagent. Then we used *Agrobacterium*-mediated method to transfer the plasmids containing the targeting expression cassettes into the prepared protocorms. The EHA105 *A. tumifaciens* strain was provided by Takara Biomedical Technology and the pCambia1301-35SN is kindly provided by Shanghai Institute of Plant Physiology and Ecology, Chinese Academy of Sciences. The transformation protocol used for the Dendrobium orchid was developed by previous study (Men et al., 2003; da Silva et al., 2011; Hsing et al., 2016; Phlaetita et al., 2015; da Silva et al., 2016).

### Tissue Culture Media

To optimize the growth of the genetically modified plants, different media were used at different stages throughout the experiment (see detailed information about the media in the Supplemental Table 1). The medium Z1 used for seed germination is based on the MS basic medium (Murashige and Skoog, 1962; Robinson et al., 2009). The medium B3-3 used for proliferation of protocorms, the co-culturing medium G and the reproduction-screening medium B3-TS are all based on the basic medium B5 (Gamborg et al., 1968), which contains mineral salts, sucrose, the B-vitamins, and 2,4-dichlorophenoxyacetic acid (1 mg/L) (Robinson et al., 2009). There are two screening media for the positive transformants: The first stage screening medium SH4-TS is different from the SH basic medium (Reil and Berger, 1996; Schenk and Hildebrandt, 1972) and the second stage screening medium FD5-TS is derived from the MS basic medium, but the content of every component is half of the original MS medium, so this basic medium is also called 1/2 MS. The rooting medium SB2-TS used for rooting of the screened positive plants is based on the B5 basic medium.

### Plant Materials

**FIGURE 2:**
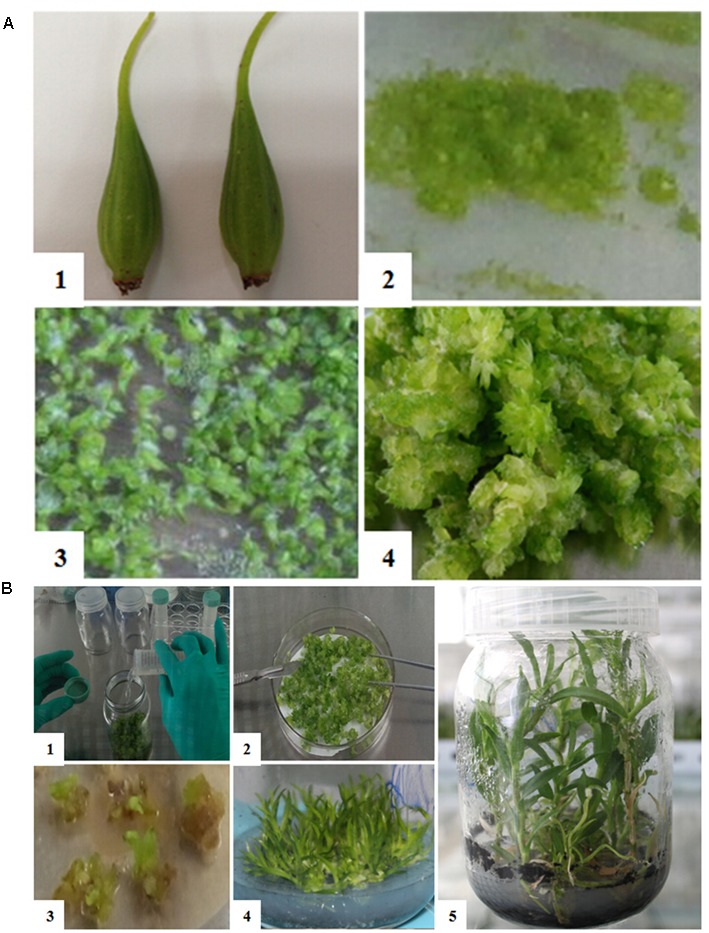
**Plant tissues preparation. (A)** The seed germination process. (1) Capsule; (2) 15 days after germination; (3) 45 days after germination; (4) The protocorms 90 days after germination. **(B)** The transgene process. (1–2) Inoculation and co-cultivation with *Agrobacterium*; (3) Selection of transgenic protocorms; (4) Differentiation and regeneration; (5) Roots after 60 days.

Mature *D. officinale* capsules were sterilized with 75% ethyl alcohol, 0.1% (w/v) mercuric chloride, then washed with sterile distilled water. After sterilization, a hole was cut at one end of the treated capsule, and seeds were sown on the Z1 medium by holding the capsule with tweezers at the other end and shaking it slightly (**Figure 2A1–3**). All cultures were maintained in an environmentally controlled room with a 12-h photoperiod at 2000Lx provided by cool white fluorescent lights. The day/night temperatures were 25 ± 3°C. In general, protocorms have been used as targets because of their higher regeneration capacity (Kanchanapoon et al., 2012). So after the seeds sprouted and developed into the protocorm stage, the protocorms were cut into small pieces around 3–5 mm in diameter and put on the B3-3 medium to produce more protocorms (**Figure 2A4**). All procedures were performed in a sterilized environment.

### Inoculation and Co-cultivation with *Agrobacterium*

The constructed vectors were transferred into the competent *Agrobacterium* cells by electroporation method. The *Agrobacterium* with vectors was inoculated in the YM liquid suspension medium with kanamycin, and incubated on a shaker at 220 rpm, at 28°C for 12 h until the density of OD600 was between 0.6 and 0.8. Next, the *Agrobacterium* cells were collected by centrifugation at 4000 rpm for 12 min at 4°C and resuspended in a moderate B5 liquid medium (Gamborg et al., 1968) to a final density of OD600 = 0.6∼0.8. The resuspended medium was the same as the basic medium, which is used for proliferation of protocorms, co-culturing and reproduction-screening. The prepared protocorms (0.5 cm in diameter) were mixed with a 50 ml bacteria liquid resuspension medium containing 100 μmol/L AS (acetosyringone) (Dutt and Grosser, 2009), which can promote exogenous gene transformation (**Figure 2B1**). Then, the mixtures were incubated on a shaker at 110 rpm for 40 min at 28°C to keep the protocorms in close and constant contact with *Agrobacterium*. After shaking, the protocorms were put on the co-cultivation medium G (**Figure 2B2**).

### Selection of Transgenic Protocorms and Plant Regeneration

After three days of co-cultivation, the infected protocorms were transferred to the B3-TS medium and incubated for 30 days, and then transferred from B3-TS medium to the SH4-TS medium to incubate for 2 weeks. We cut the protocorms into small pieces of 3–5 mm in diameter. These green tissues growing on SH4-TS medium were picked up, and then transferred to the FD5-TS medium to incubate for 30 days. Finally, the surviving plantlets growing on the FD5-TS medium were put on SB2-TS for 60 days for rooting (**Figure 2B3–5**). The conditions for all of the above cultures was as follows: temperature of 25 ± 3°C, illumination time of 12 h, illumination intensity of 2000lx, cool white fluorescent light.

### GUS Histochemical Assay

The *GUS* reporter gene encodes the β-glucuronidase, which is a hydrolase that catalyzes the cleavage of a wide variety of β-glucuronides. The transformed plants with *GUS* expression were stained with 5-bromo-4-chloro-3-indoyl β-D-glucuronide (X-Gluc). After being co-cultured with the *Agrobacterium* containing the vector pCAMBIA1301-35SN, the protocorms were immersed in X-Gluc solution (Jefferson et al., 1987) for 10 min in a mild vacuum, and then incubated overnight at 37°C. The positive transgenic protocorms should turn blue after staining.

### PCR Test for Overexpression and Genome Editing

In order to confirm whether the exogenous genes for overexpression have been successfully integrated into the plant genome, a polymerase chain reaction (PCR) is needed. Total DNA are isolated from 100 mg seedling leaves of different transformants using the GenElute^TM^ Plant Genomic DNA Miniprep Kit (Sigma-Aldrich, St. Louis, USA). The primers used in our experiment are shown in **Table 2**. First, we used the *HygR* primers to screen the DNA from positive transformants by PCR, which can confirm that the marker cassette has been transfected into tranformants successfully. Then we screened the DNA with *SG* primers, which can prove that the expression cassettes have integrated into the genome of positive transformants. Finally, in order to prove that the results are not due to PCR failure, we used the actin primer (**Table 2**) and *SG* primer to test the negative samples and positive samples. The PCR program was set as initial denaturation at 95°C for 5 min followed with 35 cycles of three-step cycle: denaturation at 95°C for 30 s, annealing at 62°C for 60 s and extension at 72°C for 40 s.

**Table 2:** The primers of the *HygR, SG*, and actin used in PCR amplification.

Gene	Function	Former primer
*HygR*	Hygromycin Marker	F: ACGCGTCGACATGTCTAAGGGCGAGGAACTC R: GGACTAGT TTATTTATAGAGTTCGTCCAT

*SG*	Superfolder green fluorescent protein, the overexpression of interested target gene	F: ATGTCTAAGGGCGAGGAA R: TTTATAGAGTTCGTCCATG

actin	The actin primer of *D. officinale*	F: GGAATGGTTAAGGCTGGATT R: CGATGGGATATTTCAAGGTG

*Note: F means the forward primer and R means the reverse primer.*

We designed the PCR primers of each target gene of CRISPR/Cas9 (**Table 3**) to screen the transformants. The forward primer annealed to the upstream sequence of the target area and the reverse primer annealed to the downstream sequence. We extracted the total DNA of the transformants to use as the template to perform PCR amplification with the primers shown in **Table 3**. The PCR products were sequenced and compared with the target gene sequences to calculate the mutagenic efficiency. Because the *HygR* expression cassette is fused with CPISPR/Cas9 expression cassettes, we could use *HygR* as a marker to detect whether the CPISPR/Cas9 expression cassettes inserted into the *D. officinale* genome by PCR amplification of total DNA of transformants. Using the same method as mentioned above, the *HygR* sequences were amplified by using the *HygR* primer shown in **Table 2**.

**Table 3:** The PCR primers of each target gene.

Gene name	Paired primers
*C3H*	F: ATTAATCAAACTTGAGCCGAA R: AGTCCGATAATAGTATCCTC

*C4H*	F: CACGGAGTTACTTACCTACCAC R: TCTTGCGCCAATGTTCACCGTA

*4CL*	F: CTCATCATCGCCGACTCCC R: CATTATCACCACACTCGCCTT

*CCR*	F: ATGGCTATAAATACGHCGCTTC R: CATGACTCATCAACGACCAC

*IRX*	F: GCTCCATCTCCTCTTTACCAA R: CTGCCTCCTGATGACCAAG

### Fluorescence Detection

The fluorescence protein *SG* is used as the marker to confirm whether an exogenous gene is expressed successfully in *D. officinale*. By observing the fluorescence emission, we can roughly detect the expression of the protein. Twenty positive *SG* transformants and one negative control were tested through observing blue light emission under fluorescence microscopy using a Zeiss Discovery V12 microscope equipped with a Zeiss Rhodamine cube KSC 295–815D with excitation wave length at 485 nm. The units of different anatomical levels from whole leaves to cells were detected under the fluorescent microscope. Images were collected using an Optronics digital camera system (Nikon D60) with manual exposure settings. For all materials, the images under bright field were recorded using the same exposure times (Zhang et al., 2015).

### Sequencing Mutants of Genome Editing

After obtaining the total DNA, the target locus of sgRNAs were amplified with the primers shown in **Table 3**. The positive PCR products were sequenced and aligned with the target gene sequence. Those samples with mutated sequences were selected for further analysis.

## Results

### GFP Overexpression

**FIGURE 3:**
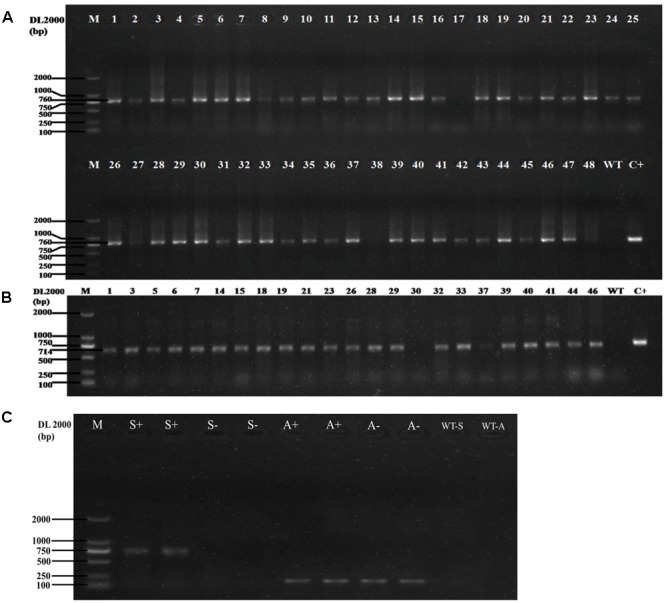
**(A)** The PCR results of the *HygR* marker of 48 transformants. The length of PCR products is 760bp, the positive rate is 93.75% (45 are positive in 48 samples). M: Marker DL2000; WT: Negative Control; C+: Positive Control. **(B)**: The PCR result of the *SG* in the transformants. The length of PCR products is 714 bp, the positive rate is 91.3% (21 are positive in 23 samples). M: Marker DL2000; WT: Negative Control; C+: Positive Control. **(C)** The PCR result of the negative and positive samples tested by the *SG* primer and actin primer. M: Marker DL2000; S+: positive samples with *SG* primer; S-: negative samples with *SG* primer; A+: positive samples with actin primer; S-: negative samples with actin primer; WT-S: *SG* primer without DNA template; WT-A: actin primer without DNA template.

In the vector of overexpression, the transcription of the *GUS* gene and the *HygR* gene is driven by the 35S promoter and ended by the 35S′ terminator. We chose 48 transformants to perform PCR amplification with *HygR* primers. The results (**Figure 3A**) showed that 45 of them were positive. Then PCR was conducted using DNA from 23 of the positive samples as templates with *SG* gene primers. The result is shown in **Figure 3B**, 91.3% (21 in 23 samples) of the candidates are the positive transformants with 714bp amplicons, which have the same length as the *SG* coding sequence. To exclude any false negative due to failure in PCR, we chose two negative samples and two positive samples to perform the PCR with SG and actin primer. As shown in **Figure 3C**, the SG band of amplification can be observed only in positive samples while the actin band of amplification can be observed in both positive and negative samples, which confirmed that those negative samples are true negatives. The PCR result confirms that the gene *SG* has been successfully inserted into the genome of *D. officinale* and the *Agrobacterium*-mediated gene transformation can work efficiently.

**FIGURE 4:**
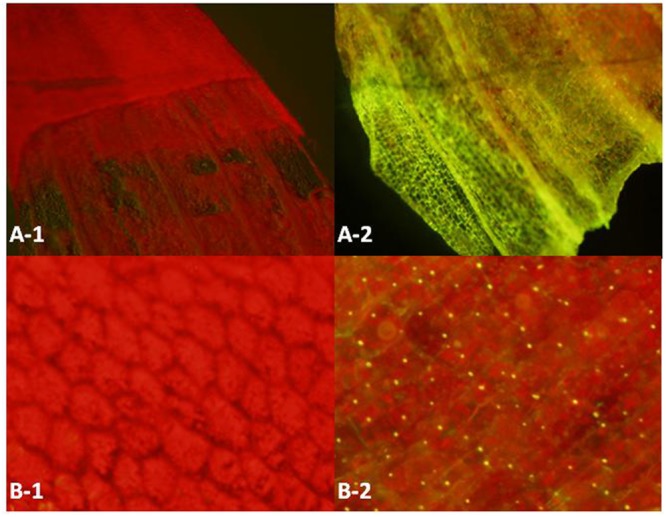
**Fluorescence emission of different anatomical levels (A,B)** of the negative controls (1) and test samples (2). The excitation light is blue light (485 nm) and the fluorescence light is the mixture of red light (photosynthetic pigments, 625∼740 nm) and green light (superfolder green fluorescent protein gene, 500∼565 nm). Annotation: A: Epidermis; B: Mesophyll cell.

Superfolder green fluorescent protein (*SG*) is a well-folded variant of green fluorescent protein (Pedelacq et al., 2006), which can emit green light under the excitation light with wavelength at 485nm. The fluorescent signals at different anatomical levels from whole leaves to cells were detected under a fluorescent microscope. According to **Figure 4**, because of the existence of photosynthetic pigments that can emit red light under the excitation light, the controls and the samples all have strong red fluorescence background. When superfolder green fluorescent protein co-localizes with the red fluorescent pigment, a yellow spot will show in the merged image. Compared with the controls, all of the 20 samples coming from the *SG* transformants showed the expression of superfolder green fluorescent protein gene (data not shown). This result confirms that the transformed genes can transcribe and translate into functional proteins, and the established transformation system in *D. officinale* can work efficiently.

### Testing of Promoters by Comparing Early Expression of *GUS* Gene 3 Days after Transformation

**FIGURE 5:**
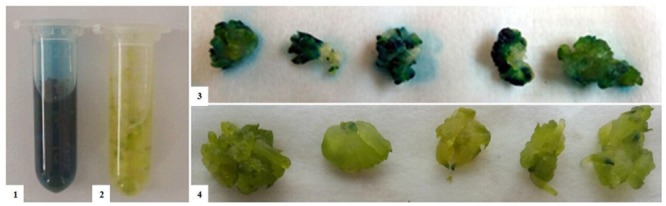
***GUS* histochemical assay result of *D. officinale*.** (1,3): All of the samples turn blue, which indicates that the expression cassettes have transformed into the cells and work well. (2,4): In the negative control, which are untransformed tissue, no sample turns blue.

We used the GUS gene as a reporter to screen the positive transformants at an early stage after transformation. The protocorm tissues, which were successfully transfected with transgene expression cassettes, can be stained to blue with X-Gluc. As shown in **Figure 5**), when treated with X-Gluc, the *Agrobacterium* infected protocorms turn blue in some areas. The early expression of *GUS* indicates that the expression cassettes have been transformed into the protocorm cells.

**FIGURE 6:**
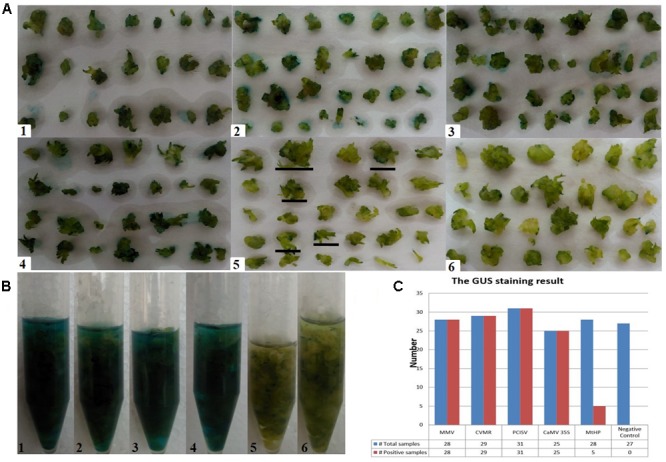
**Testing the relative strength of five constitutively active promoters. (A–B)** The *GUS* histochemical assay result. (1-4) For promoters MMV, CVMR, PCISV, and CaMV 35S, all samples turn blue; (5) for promoter MtHP, part of the samples turn blue; (6) for the negative control, which is untransformed tissue, no sample turns blue. **(C)** The GUS staining results statistics. Total samples: The total number of test samples; Positive samples: The number of positive samples.

To identify efficient promoters, the four promoters MtHP, CVMV, MMV, and PCISV were tested and the results were compared with the 35S promoter. The results are shown in **Figure 6**. The positive ratio of each promoter was calculated based on examining each of the protocorms in **Figure 6A** in different directions under a microscope, shown in **Table 4**. The results showed that MMV, CVMV, and PCISV are as effective as the 35S promoter with strong expression in all protocorms examined, while MtHP is less effective than the 35S promoter. Because only tiny spots on protocorms, and not whole protocorms, were stained blue from GUS transformants driven by MtHP, it is not easy to differentiate them from negative controls based on the picture in **Figure 6**. However, MtHP still showed weak expression in 17.8% of the protocorms probably due to MtHP promoter leakage in the orchid. Our results proved that MMV, CVMV, and PCISV can be used as efficient promoters in the transgenic system of *D. officinale*.

**Table 4:** The *GUS* histochemical assay result.

Promoter	Sample amount	Positive number	Positive rate (%)
MMV	22	22	100
CVMR	29	29	100
PCISV	31	31	100
CaMV 35S	26	26	100
MtHP	28	5	17.8

### CRISPR-Cas9 Mediated Genome Editing

**FIGURE 7:**
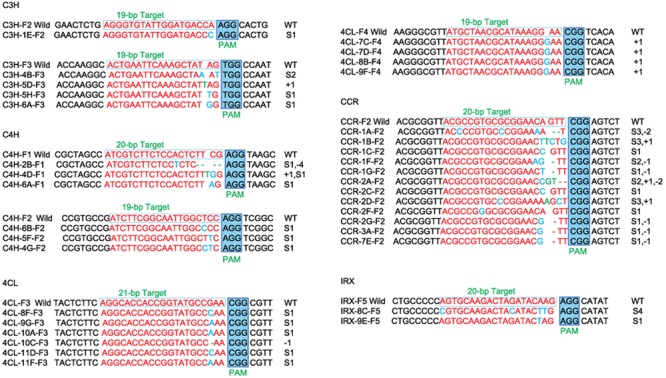
**The mutation locations at the chosen targets and their downstream sequences.** Here, some samples failed during the sequencing which are not shown. Only the positive results are shown. *CH3*: 2 target loci (F2, F3) have been sequenced, *CH4*: 2 target loci (F1, F2), *4CL*: 2 target loci (F2, F3), *CCR*: 1 target locus (F1), *IRX*: 1 target locus (F2). The red portions of the wilds and samples are the target locus, the blue block is the PAM region, and the change in the number of nucleotides is shown in blue (substitution) and green (insertion). The “S” means the number of substitutions; the “+” means the number of insertions; the “-” means the number of deletions.

Here we chose five genes as the targets to explore the functional CRISPR/Cas9 system in *D. officinale*. Because the *HygR* gene is fused with the CRISPR/Cas9 expression cassettes, it can be used as a marker to detect the rate of successful insertion of CRISPR/Cas9 expression cassettes into transformants’ genomes. We randomly chose 47 samples to demonstrate the positive rate. As shown in Supplemental Figure 1, 93.6% of the all detected transformants are positive plants (44 out of 47 samples), and only three transformants are negative plants or reflect weak positive characteristics. To further check whether the CRISPR/Cas9 system can edit the target genes in the host cells, the target loci were PCR amplified with the primers shown in **Table 3** and then sequenced. We analyzed the sequencing result of each target gene to characterize the mutation introduced by using CRISPR/Cas9. The results are shown in **Table 5** and **Figure 7**. The original sequencing results are shown in supplemental Figure 2.

**Table 5:** Ratios of mutation types at the different target sites of five genes in mutant plants.

Target gene	Site	No. of examined lines	No. of lines with mutations	Deletion	Insertion	Substitution	Insertion and Substitution	Deletion and Substitution	Insertion, Deletion and Substitution	Mutation rate (%)	Average rate (%)
*C3H*	sgRNA1	10	0	0	0	0	0	0	0	0	16.7
	sgRNA2	10	1	0	0	1	0	0	0	0.1	
	sgRNA3	10	4	0	1	3	0	0	0	0.4	
*C4H*	sgRNA1	10	3	0	0	1	1	1	0	0.3	20
	sgRNA2	10	3	0	0	3	0	0	0	0.3	
	sgRNA3	10	0	0	0	0	0	0	0	0	
*4CL*	sgRNA1	10	0	0	0	0	0	0	0	0	33.3
	sgRNA2	10	6	1	0	5	0	0	0	0.6	
	sgRNA3	10	4	0	4	0	0	0	0	0.4	
*CCR*	sgRNA1	10	10	0	0	3	2	4	1	1	33.3
	sgRNA2	10	0	0	0	0	0	0	0	0	
	sgRNA3	10	0	0	0	0	0	0	0	0	
*IRX*	sgRNA1	10	0	0	0	0	0	0	0	0	6.7
	sgRNA2	10	2	0	0	2	0	0	0	0.2	
	sgRNA3	10	0	0	0	0	0	0	0	0	

*The mutation rate is based on the number of mutation type lines out of the total number of all examined lines.*

As shown in **Figure 7**, the sequences of each gene from the corresponding samples confirm lesions in target regions. There are two mutated loci (F2, F3) in *C3H* gene: the first locus (F2) has nucleotide substitution in one sample; the second locus (F3) has nucleotide substitution in three samples and nucleotide insertion in one sample. Two mutated target loci (F1, F2) were found in *C4H* gene. For F1 locus, we found nucleotide substitution and nucleotide deletion occurred in the first sample simultaneously. Nucleotide substitution and nucleotide insertion occurred in the second sample simultaneously, and nucleotide substitution occurred in the third sample. For F2 locus, nucleotide substitution occurred in all three samples. In *4CL* gene, we detected two mutated target loci (F2, F3). Nucleotide substitution in five samples and nucleotide deletion in one sample occurred in locus F2. The F3 locus has nucleotide substitution in four samples. Only one mutated target locus was found in both *CCR* and *IRX*. In *CCR* gene, nucleotide substitution, insertion or deletion occurred in ten samples, while in *IRX* gene, nucleotide substitution occurred in two samples. The percentages of mutations are shown in **Table 5**, which varied between 10 and 100%.

These results provided evidence that the established CRISPR/Cas9 system can work efficiently although the phenotype of the mutant plants developed in this study has not been checked yet due to the slow growth of *D. officinale.* We can further use this system to modify other pathways to study the effects of gene functions on the development and metabolism of *D. officinale*.

## Discussion

As the second largest family of flowering plants, many gene transformation studies in orchids have been reported and lots of novel phenotypes have been obtained such as virus resistance (Chen and Lin, 2011) and abnormal multiple shoot development (Yu et al., 2001). Only a few studies have used the RNAi strategy, such as to reduce the target gene expression to modify floral patterns in *Dendrobium* Sonia (Ratanasut et al., 2015) or to generate a semi-dwarf phenotype and brilliant green leaves in *Oncidium hybrid* orchid (Liu et al., 2014). The RNAi-mediated approach had several limitations such as incomplete loss-of-function and extensive off-target activities in many plant species although the off-target activities have not been examined in orchids systematically (Xu et al., 2006). While RNAi is capable of down-regulating the transcription activity of target gene, CRISPR/Cas9 is able to directly edit the genome sequence of target gene with high accuracy and relatively low off-target activities (Barrangou et al., 2015). This genome editing system was discovered in bacteria (Horvath and Barrangou, 2010; Marraffini and Sontheimer, 2010) and then quickly applied in other model organisms (Hwang et al., 2013; Jiang et al., 2013; Hai et al., 2014; Liang et al., 2014; Shan et al., 2014; Shao et al., 2014; Chen et al., 2015; Jacobs et al., 2015). However, the CRISPR/Cas9 system has never been applied in orchids successfully. Here, we applied the CRISPR/Cas9 system for genome editing using the *Agrobacterium* mediated transformation in *D. officinale*. Our results have shown that this CRISPR/Cas9 system can introduce mutations into the targets accurately. This will be a convenient platform to uncover the gene functions of and create novel cultivars in *D. officinale*, and maybe also in other orchids. Comparing the efficiency of different targets in every candidate gene, we found that these target sites have different mutation rates and some targets totally fail to be mutated. A possible reason may be the difference in higher chromatin structure of those target sites. Some target sites may be densely folded on the chromosome while others may have a relatively loose form leading to different accessibility to the Cas9 protein and sgRNA complex. Further study on the higher level structure of those target sequences could help to explain the difference in mutation rates. Moreover, we still need more work to get quantitative data on the efficiency of each step in order to fine-tune this system and obtain optimal performance. For example, we can use the T7E1 or PCR/RE assay to detect potential mutated events and evaluate the genotypes of mutants (Shan et al., 2014; Zhang et al., 2014), apply the transient expression to introduce mutations at the targets without integration of exogenous gene cassettes (Zhang Y. et al., 2016), and analyze the off-target sites (Feng et al., 2014). The final goal of CRISPR/Cas9 applications is to precisely mutate target genes and obtain a homozygous line in one generation (Li et al., 2015; Svitashev et al., 2015).

## Conclusion

Here we have successfully identified efficient promoters for exogenous gene overexpression and applied the CRISPR/Cas9 system to edit endogenous genes in *D. officinale*. These results proved that a genetic manipulation tool for horticultural, medicinal or other applications is available for *D. officinale*. In future research, we will improve our protocol and add more genetic tools to the toolbox of genetic manipulation for *D. officinale* with the aim of creating an efficient model for the genetic investigation and molecular breeding of orchids.

## Author Contributions

WW, JC, and YD designed the study. LK, SH, and CZ completed the experiments, LK and HC wrote the manuscript, WZ, ZX, YZ, JC, PZ, and GZ edited the manuscript, LY, FH, JC, and SY provided advice on editing the manuscript. All authors read and approved the final manuscript.

## Conflict of Interest Statement

There is a patent pending for the CRISPR/Cas9 system we used in this research (Zhang and Zhang, 2015).

The reviewer AM and handling Editor declared their shared affiliation, and the handling Editor states that the process nevertheless met the standards of a fair and objective review.
