# Corrigendum: Building a Genetic Manipulation Tool Box for Orchid Biology: Identification of Constitutive Promoters and Application of CRISPR/Cas9 in the Orchid, *Dendrobium officinale*

**DOI:** 10.3389/fpls.2017.00664

**Published:** 2017-04-25

**Authors:** Ling Kui, Haitao Chen, Weixiong Zhang, Simei He, Zijun Xiong, Yesheng Zhang, Liang Yan, Chaofang Zhong, Fengmei He, Junwen Chen, Peng Zeng, Guanghui Zhang, Shengchao Yang, Yang Dong, Wen Wang, Jing Cai

**Affiliations:** ^1^State Key Laboratory of Genetic Resources and Evolution, Kunming Institute of Zoology, Chinese Academy of SciencesKunming, China; ^2^Kunming College of Life Science, University of Chinese Academy of SciencesKunming, China; ^3^NowbioKunming, China; ^4^State Key Laboratory of Quality Research in Chinese Medicine, Institute of Chinese Medical Sciences, University of MacauMacau, China; ^5^National and Local Joint Engineering Research Center on Gemplasm Utilization and Innovation of Chinese Medicinal Materials in Southwest China, Yunnan Agricultural UniversityKunming, China; ^6^China National GeneBank, BGI-ShenzhenShenzhen, China; ^7^Pu'er Institute of Pu-er TeaPu'er, China; ^8^Key Laboratory of Molecular Biophysics of the Ministry of Education, College of Life Science and Technology, Huazhong University of Science and TechnologyWuhan, China; ^9^College of Horticulture and Landscape, Yunnan Agricultural UniversityKunming, China; ^10^Faculty of Life Science and Technology, Kunming University of Science and technologyKunming, China; ^11^Province Key Laboratory, Biological Big Data College, Yunnan Agricultural UniversityKunming, China; ^12^Shenzhen Orchid Conservation and Research CenterShenzhen, China

**Keywords:** *Dendrobium officinale*, transgene, vector construction, CRISPR/Cas9 gene editing, overexpression, lignocellulose biosynthesis, gene promoters

**Addition of Affiliation**

In the published article, there was an error regarding the affiliation for Ling Kui. As well as having Affiliation 1, they should also have Kunming College of Life Science, University of Chinese Academy of Sciences, Kunming, China. The authors apologize for this error and state that this does not change the scientific conclusions of the article in any way.

**Missing Funding**

In the original article, we neglected to include the funder Guizhou Academy of Tobacco Science (Project “The genome and transcriptome analysis of various plants in Nicotiana.”), Y205941151 to WW. The authors apologize for this error and state that this does not change the scientific conclusions of the article in any way.

## Conflict of interest statement

The authors declare that the research was conducted in the absence of any commercial or financial relationships that could be construed as a potential conflict of interest.

