# Supplementary material for: Building a Genetic Manipulation Tool Box for Orchid Biology: Identification of Constitutive Promoters and Application of CRISPR/Cas9 in the Orchid, Dendrobium officinale
Source: Front Plant Sci. 2017 Jan 12;7:2036. doi: 10.3389/fpls.2016.02036 (PMC5226938; doi:10.3389/fpls.2016.02036)
Supplement: Supplementary file 1 [file Presentation_1.PDF]

Establishing an efficient transgenic system and CRISPR/Cas9-mediated targeted mutagenesis in *Dendrobium officinale*

Ling Kui[1†], Haitao Chen[1,2†], Simei He[3], Zijun Xiong[4], Yesheng Zhang[1,2], Liang Yan[5], Chaofang Zhong[6], Fengmei He[7], Guanghui Zhang[3], Yang Dong[2,8,9], Jing Cai[10*] and Wen Wang[1*]

*** Correspondence: Wen Wang:** wwang@mail.kiz.ac.cn **Jing Cai:** jingcai@umac.mo.

Supplemental material

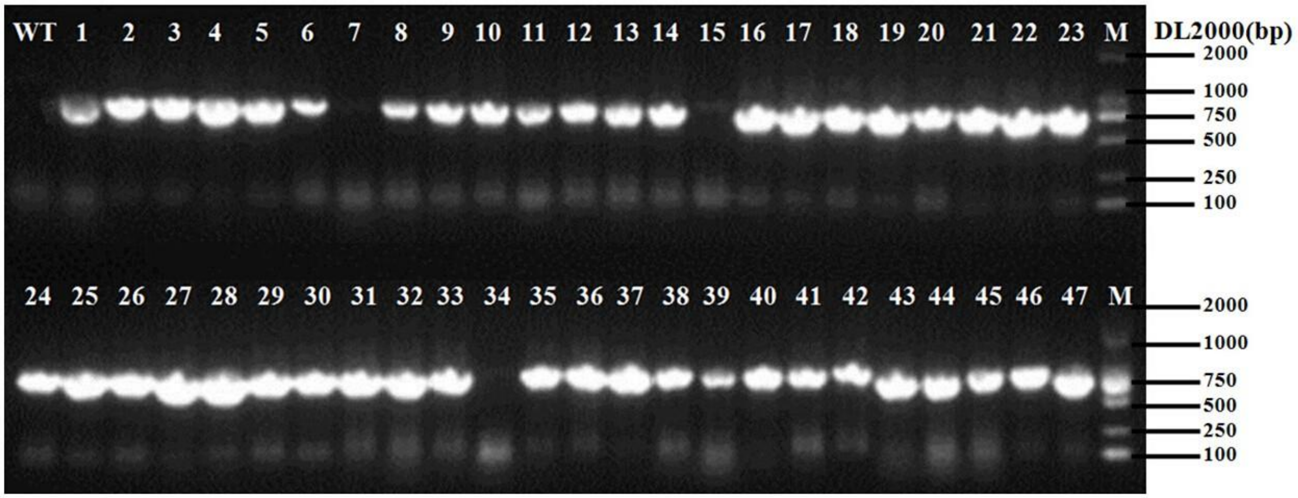

Figure 1: The PCR results of *HygR* marker in gene knock-out test. The PCR results of *HygR* marker of 47 transformants, the positive rate is 93.6%. M: Marker DL2000; WT: Negative Control.

C3H

```
C3H-F1_Wild  TTTCAGGTGTGGCTTTCAACAACATAACAAGGCTTGCATTTGGAAAACGTTTTGTGAACTCTGAGGGTGTATTGGATGACCAAGGCACTGAATTCAAAGCT
C3H-2G_F1    TTTCAGGTGTGGCTTTCAACAACATAACAAGGCTTGCATTTGGAAAACGTTTTGTGAACTCTGAGGGTGTATTGGATGACCAAGGCACTGAATTCAAAGCT
C3H-2H_F1    TTTCAGGTGTGGCTTTCAACAACATAACAAGGCTTGCATTTGGAAAACGTTTTGTGAACTCTGAGGGTGTATTGGATGACCAAGGCACTGAATTCAAAGCT
C3H-3A_F1    TTTCAGGTGTGGCTTTCAACAACATAACAAGGCTTGCATTTGGAAAACGTTTTGTGAACTCTGAGGGTGTATTGGATGACCAAGGCACTGAATTCAAAGCT
C3H-3B_F1    TTTCAGGTGTGGCTTTCAACAACATAACAAGGCTTGCATTTGGAAAACGTTTTGTGAACTCTGAGGGTGTATTGGATGACCAAGGCACTGAATTCAAAGCT
C3H-3C_F1    TTTCAGGTGTGGCTTTCAACAACATAACAAGGCTTGCATTTGGAAAACGTTTTGTGAACTCTGAGGGTGTATTGGATGACCAAGGCACTGAATTCAAAGCT
C3H-3D_F1    TTTCAGGTGTGGCTTTCAACAACATAACAAGGCTTGCATTTGGAAAACGTTTTGTGAACTCTGAGGGTGTATTGGATGACCAAGGCACTGAATTCAAAGCT
C3H-3E_F1    TTTCAGGTGTGGCTTTCAACAACATAACAAGGCTTGCATTTGGAAAACGTTTTGTGAACTCTGAGGGTGTATTGGATGACCAAGGCACTGAATTCAAAGCT
C3H-3F_F1    TTTCAGGTGTGGCTTTCAACAACATAACAAGGCTTGCATTTGGAAAACGTTTTGTGAACTCTGAGGGTGTATTGGATGACCAAGGCACTGAATTCAAAGCT
C3H-3G_F1    TTTCAGGTGTGGCTTTCAACAACATAACAAGGCTTGCATTTGGAAAACGTTTTGTGAACTCTGAGGGTGTATTGGATGACCAAGGCACTGAATTCAAAGCT
C3H-3H_F1    TTTCAGGTGTGGCTTTCAACAACATAACAAGGCTTGCATTTGGAAAACGTTTTGTGAACTCTGAGGGTGTATTGGATGACCAAGGCACTGAATTCAAAGCT
                                                    PAM

C3H-F2_Wild  AACAACATAACAAGGCTTGCATTTGGAAAACGTTTTGTGAACTCTGAGGGTGTATTGGATGACCAAGGCACTGAATTCAAAGCTATAGTGGCCAATGGCCT
C3H-1E_F2    AACAACATAACAAGGCTTGCATTTGGAAAACGTTTTGTGAACTCTGAGGGTGTATTGGATGACCCAGGCCCTGAATTCCAAGCTATAATGGCCAATGGCCT
C3H-1A_F2    AACAACATAACAAGGCTTGCATTTGGAAAACGTTTTGTGAACTCTGAGGGTGTATTGGATGACCAAGGCACTGAATTCAAAGCTATAGTGGCCAATGGCCT
C3H-1B_F2    AACAACATAACAAGGCTTGCATTTGGAAAACGTTTTGTGAACTCTGAGGGTGTATTGGATGACCAAGGCACTGAATTCAAAGCTATAGTGGCCAATGGCCT
C3H-1C_F2    AACAACATAACAAGGCTTGCATTTGGAAAACGTTTTGTGAACTCTGAGGGTGTATTGGATGACCAAGGCACTGAATTCAAAGCTATAGTGGCCAATGGCCT
C3H-1D_F2    AACAACATAACAAGGCTTGCATTTGGAAAACGTTTTGTGAACTCTGAGGGTGTATTGGATGACCAAGGCACTGAATTCAAAGCTATAGTGGCCAATGGCCT
C3H-1F_F2    AACAACATAACAAGGCTTGCATTTGGAAAACGTTTTGTGAACTCTGAGGGTGTATTGGATGACCAAGGCACTGAATTCAAAGCTATAGTGGCCAATGGCCT
C3H-1G_F2    AACAACATAACAAGGCTTGCATTTGGAAAACGTTTTGTGAACTCTGAGGGTGTATTGGATGACCAAGGCACTGAATTCAAAGCTATAGTGGCCAATGGCCT
C3H-1H_F2    AACAACATAACAAGGCTTGCATTTGGAAAACGTTTTGTGAACTCTGAGGGTGTATTGGATGACCAAGGCACTGAATTCAAAGCTATAGTGGCCAATGGCCT
C3H-2A_F2    AACAACATAACAAGGCTTGCATTTGGAAAACGTTTTGTGAACTCTGAGGGTGTATTGGATGACCAAGGCACTGAATTCAAAGCTATAGTGGCCAATGGCCT
C3H-2B_F2    AACAACATAACAAGGCTTGCATTTGGAAAACGTTTTGTGAACTCTGAGGGTGTATTGGATGACCAAGGCACTGAATTCAAAGCTATAGTGGCCAATGGCCT
                                                                                 PAM

C3H-F3_Wild  TTGCATTTGGAAAACGTTTTGTGAACTCTGAGGGTGTATTGGATGACCAAGGCACTGAATTCAAAGCTAT-AGTGGCCAATGGCCTCAAATTTGGCGCATC
C3H-4B_F3    TTGCATTTGGAAAACGTTTTGTGAACTCTGAGGGTGTATTGGATGACCAAGGCACTGAATTCAAAGCTAA-ATGGCCCAAGGGCCCCAAATTTGCCCCCTC
C3H-5D_F3    TTGCATTTGGAAAACGTTTTGTGAACTCTGAGGGTGTATTGGATGACCAAGGCACTGAATTCAAAGCTATTAGTGGCCAATGGCCTCAAATTTGGCGCATC
C3H-5H_F3    TTGCATTTGGAAAACGTTTTGTGAACTCTGAGGGTGTATTGGATGACCAAGGCACTGAATTCAAAGCTAT-TGGGGCCCGGGGCCCCAAATTTGGCCCCTC
C3H-6A_F3    TTGCATTTGGAAAACGTTTTGTGAACTCTGAGGGTGTATTGGATGACCAAGGCACTGAATTCAAAGCTAT-GGTGGCCAATGGCCTCCAATTTGGCCCCTC
C3H-4F_F3    TTGCATTTGGAAAACGTTTTGTGAACTCTGAGGGTGTATTGGATGACCAAGGCACTGAATTCAAAGCTAT-AGTGGCCAATGGCCTCAAATTTGGCGCATC
C3H-4C_F3    TTGCATTTGGAAAACGTTTTGTGAACTCTGAGGGTGTATTGGATGACCAAGGCACTGAATTCAAAGCTAT-AGTGGCCAATGGCCTCAAATTTGGCGCATC
C3H-5C_F3    TTGCATTTGGAAAACGTTTTGTGAACTCTGAGGGTGTATTGGATGACCAAGGCACTGAATTCAAAGCTAT-AGTGGCCAATGGCCTCAAATTTGGCGCATC
C3H-4G_F3    TTGCATTTGGAAAACGTTTTGTGAACTCTGAGGGTGTATTGGATGACCAAGGCACTGAATTCAAAGCTAT-AGTGGCCAATGGCCTCAAATTTGGCGCATC
C3H-4H_F3    TTGCATTTGGAAAACGTTTTGTGAACTCTGAGGGTGTATTGGATGACCAAGGCACTGAATTCAAAGCTAT-AGTGGCCAATGGCCTCAAATTTGGCGCATC
C3H-4C_F3    TTGCATTTGGAAAACGTTTTGTGAACTCTGAGGGTGTATTGGATGACCAAGGCACTGAATTCAAAGCTAT-AGTGGCCAATGGCCTCAAATTTGGCGCATC
                                                                                    PAM
```

C4H

```
C4H-F1_Wild  CTGGAGAAAGCTCTCCTCGGTCTCTTCGCGGCGGTGACGCTAGCCATCGTCTTCTCCACTCTTC-GAGGTAAGCGATTCAAGCTTCCGCCGGGGCCGCTCCC
C4H-2B_F1    CTGGAGAAAGCTCTCCTCGGTCTCTTCGCGGCGGTGACGCTAGCCATCGTCTTCTCCTCTC-----AGGAGGGCGATTAATGCTTCTGCCGGGGGGGCTCCC
C4H-4D_F1    CTGGAGAAAGCTCTCCTCGGTCTCTTCGCGGCGGTGACGCTAGCCATCGTCTTCTCCACTCTTTGGAGGAAGGAAATTTATGCTGCGGCGGGGGCCCCTCCC
C4H-6A_F1    CTGGAGAAAGCTCTCCTCGGTCTCTTCGCGGCGGTGACGCTAGCCATCGTCTTCTCCACTCTT-AGAGGTAAGCGATTCACTCTTCCGGCGGGGCCGCTCCC
C4H-2C_F1    CTGGAGAAAGCTCTCCTCGGTCTCTTCGCGGCGGTGACGCTAGCCATCGTCTTCTCCACTCTTC-GAGGTAAGCGATTCAAGCTTCCGCCGGGGCCGCTCCC
C4H-2D_F1    CTGGAGAAAGCTCTCCTCGGTCTCTTCGCGGCGGTGACGCTAGCCATCGTCTTCTCCACTCTTC-GAGGTAAGCGATTCAAGCTTCCGCCGGGGCCGCTCCC
C4H-2G_F1    CTGGAGAAAGCTCTCCTCGGTCTCTTCGCGGCGGTGACGCTAGCCATCGTCTTCTCCACTCTTC-GAGGTAAGCGATTCAAGCTTCCGCCGGGGCCGCTCCC
C4H-2H_F1    CTGGAGAAAGCTCTCCTCGGTCTCTTCGCGGCGGTGACGCTAGCCATCGTCTTCTCCACTCTTC-GAGGTAAGCGATTCAAGCTTCCGCCGGGGCCGCTCCC
C4H-3A_F1    CTGGAGAAAGCTCTCCTCGGTCTCTTCGCGGCGGTGACGCTAGCCATCGTCTTCTCCACTCTTC-GAGGTAAGCGATTCAAGCTTCCGCCGGGGCCGCTCCC
C4H-3B_F1    CTGGAGAAAGCTCTCCTCGGTCTCTTCGCGGCGGTGACGCTAGCCATCGTCTTCTCCACTCTTC-GAGGTAAGCGATTCAAGCTTCCGCCGGGGCCGCTCCC
C4H-3C_F1    CTGGAGAAAGCTCTCCTCGGTCTCTTCGCGGCGGTGACGCTAGCCATCGTCTTCTCCACTCTTC-GAGGTAAGCGATTCAAGCTTCCGCCGGGGCCGCTCCC
                                                                                 PAM

C4H-F2_Wild  CACTCTTCGAGGTAAGCGATTCAAGCTTCCGCCGGGGCCGCTCCCCGTGCCGATCTTCGGCAATTGGCTCC-AGGTCGGCGATGATCTGAACCAGCGCAATC
C4H-6B_F2    CACTCTTCGAGGTAAGCGATTCAAGCTTCCGCCGGGGCCGCTCCCCGTGCCGATCTTCGGCAATTGGCCCCGGGGGCGAAGATTTTAAAAACCGCCAACTTC
C4H-5F_F2    CACTCTTCGAGGTAAGCGATTCAAGCTTCCGCCGGGGCCGCTCCCCGTGCCGATCTTCGGCAATTGGCTTC-CGGGCCGAGAAGAACTGAAACCGCCCCATC
C4H-4G_F2    CACTCTTCGAGGTAAGCGATTCAAGCTTCCGCCGGGGCCGCTCCCCGTGCCGATCTTCGGCAATTGGCCTCGGGGGCGGAGAAGATCAGAACCCGCACTTTC
C4H-6C_F2    CACTCTTCGAGGTAAGCGATTCAAGCTTCCGCCGGGGCCGCTCCCCGTGCCGATCTTCGGCAATTGGCTCC-AGGTCGGCGATGATCTGAACCAGCGCAATC
C4H-6D_F2    CACTCTTCGAGGTAAGCGATTCAAGCTTCCGCCGGGGCCGCTCCCCGTGCCGATCTTCGGCAATTGGCTCC-AGGTCGGCGATGATCTGAACCAGCGCAATC
C4H-6F_F2    CACTCTTCGAGGTAAGCGATTCAAGCTTCCGCCGGGGCCGCTCCCCGTGCCGATCTTCGGCAATTGGCTCC-AGGGCGGCGAAGATCTGAACCAGCGCAATC
C4H-6G_F2    CACTCTTCGAGGTAAGCGATTCAAGCTTCCGCCGGGGCCGCTCCCCGTGCCGATCTTCGGCAATTGGCTCC-AGGTCGGCGATGATCTGAACCAGCGCAATC
C4H-6H_F2    CACTCTTCGAGGTAAGCGATTCAAGCTTCCGCCGGGGCCGCTCCCCGTGCCGATCTTCGGCAATTGGCTCC-AGGTCGGCGATGATCTGAACCAGCGCAATC
C4H-7B_F2    CACTCTTCGAGGTAAGCGATTCAAGCTTCCGCCGGGGCCGCTCCCCGTGCCGATCTTCGGCAATTGGCTCC-AGGTCGGCGATGATCTGAACCAGCGCAATC
C4H-7C_F2    CACTCTTCGAGGTAAGCGATTCAAGCTTCCGCCGGGGCCGCTCCCCGTGCCGATCTTCGGCAATTGGCTCC-AGGTCGGCGATGATCTGAACCAGCGCAATC
                                                                                      PAM

C4H-F3_Wild  CTCGCCGCCCTAGCTAAAAAGTTCGGCGACATCTTCCTACTCCGAATGGGCCAGCGCAACCTCGTCGTCGTCTCGTCCCCTGATCTGGCCCGCGAGGTCCT
C4H-1B_F3    CTCGCCGCCCTAGCTAAAAAGTTCGGCGACATCTTCCTACTCCGAATGGGCCAGCGCAACCTCGTCGTCGTCTCGTCCCCTGATCTGGCCCGCGAGGTCCT
C4H-1C_F3    CTCGCCGCCCTAGCTAAAAAGTTCGGCGACATCTTCCTACTCCGAATGGGTCAGCGCAACCTCGTCGTCGTCTCGTCCCCTGATCTGGCCCGCGAGGTCCT
C4H-1D_F3    CTCGCCGCCCTAGCTAAAAAGTTCGGCGACATCTTCCTACTCCGAATGGGTCAGCGCAACCTCGTCGTCGTCTCGTCCCCTGATCTGGCCCGCGAGGTCCT
C4H-1E_F3    CTCGCCGCCCTAGCTAAAAAGTTCGGCGACATCTTCCTACTCCGAATGGGCCAGCGCAACCTCGTCGTCGTCTCGTCCCCTGATCTGGCCCGCGAGGTCCT
C4H-1G_F3    CTCGCCGCCCTAGCTAAAAAGTTCGGCGACATCTTCCTACTCCGAATGGGCCAGCGCAACCTCGTCGTCGTCTCGTCCCCTGATCTGGCCCGCGAGGTCCT
C4H-1H_F3    CTCGCCGCCCTAGCTAAAAAGTTCGGCGACATCTTCCTACTCCGAATGGGTCAGCGCAACCTCGTCGTCGTCTCGTCCCCTGATCTGGCCCGCGAGGTCCT
C4H-2F_F3    CTCGCCGCCCTAGCTAAAAAGTTCGGCGACATCTTCCTACTCCGAATGGGTCAGCGCAACCTCGTCGTCGTCTCGTCCCCTGATCTGGCCCGCGAGGTCCT
C4H-5A_F3    CTCGCCGCCCTAGCTAAAAAGTTCGGCGACATCTTCCTACTCCGAATGGGTCAGCGCAACCTCGTCGTCGTCTCGTCCCCTGATCTGGCCCGCGAGGTCCT
C4H-5B_F3    CTCGCCGCCCTAGCTAAAAAGTTCGGCGACATCTTCCTACTCCGAATGGGTCAGCGCAACCTCGTCGTCGTCTCGTCCCCTGATCTGGCCCGCGAGGTCCT
C4H-5C_F3    CTCGCCGCCCTAGCTAAAAAGTTCGGCGACATCTTCCTACTCCGAATGGGTCAGCGCAACCTCGTCGTCGTCTCGTCCCCTGATCTGGCCCGCGAGGTCCT
                                                           PAM
```

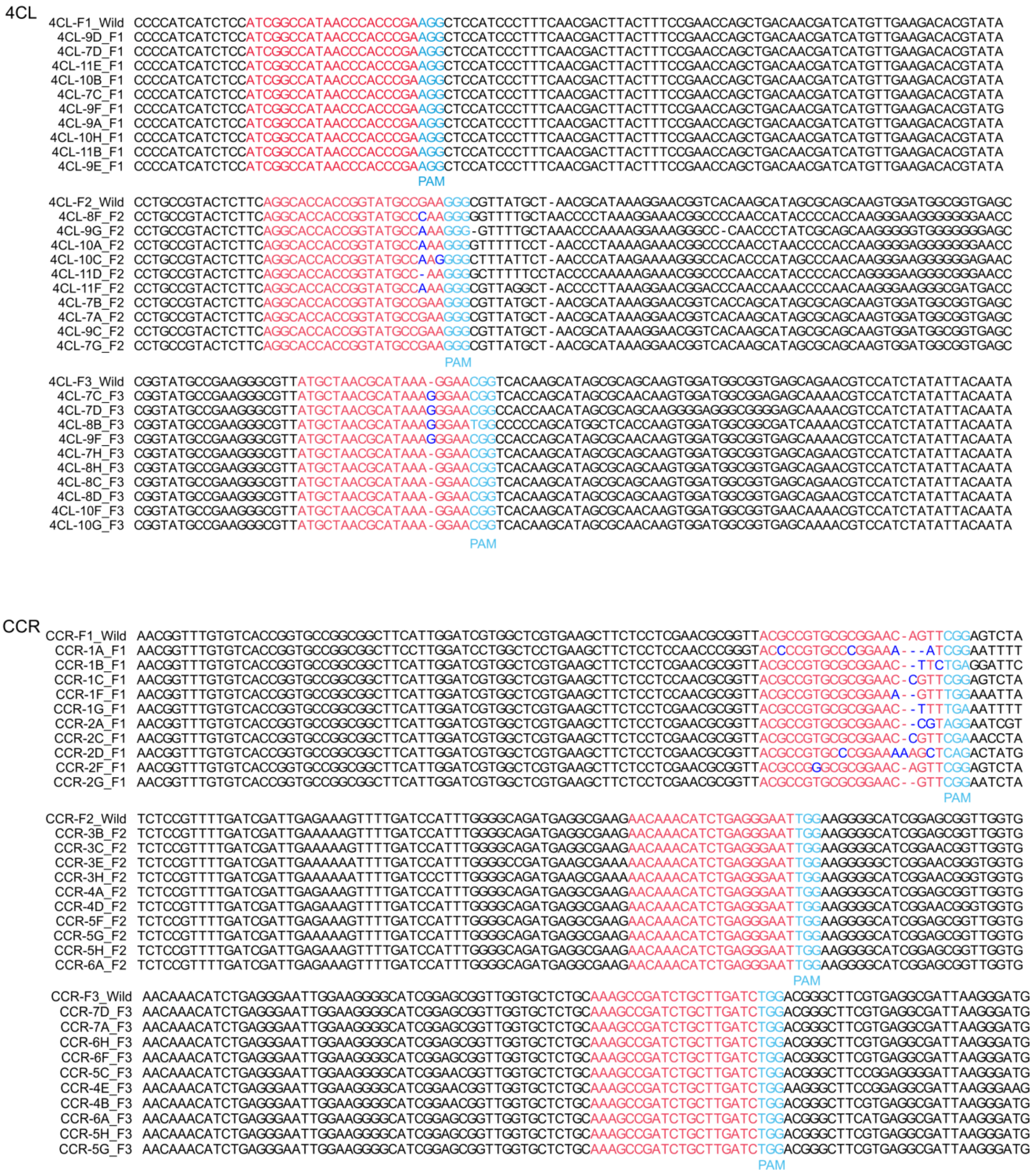
4CL
4CL-F1_Wild
4CL-9D_F1
4CL-7D_F1
4CL-11E_F1
4CL-10B_F1
4CL-7C_F1
4CL-9F_F1
4CL-9A_F1
4CL-10H_F1
4CL-11B_F1
4CL-9E_F1
PAM
4CL-F2_Wild
4CL-8F_F2
4CL-9G_F2
4CL-10A_F2
4CL-10C_F2
4CL-11D_F2
4CL-11F_F2
4CL-7B_F2
4CL-7A_F2
4CL-9C_F2
4CL-7G_F2
PAM
4CL-F3_Wild
4CL-7C_F3
4CL-7D_F3
4CL-8B_F3
4CL-9F_F3
4CL-7H_F3
4CL-8H_F3
4CL-8C_F3
4CL-8D_F3
4CL-10F_F3
4CL-10G_F3
PAM
CCR
CCR-F1_Wild
CCR-1A_F1
CCR-1B_F1
CCR-1C_F1
CCR-1F_F1
CCR-1G_F1
CCR-2A_F1
CCR-2C_F1
CCR-2D_F1
CCR-2F_F1
CCR-2G_F1
PAM
CCR-F2_Wild
CCR-3B_F2
CCR-3C_F2
CCR-3E_F2
CCR-3H_F2
CCR-4A_F2
CCR-4D_F2
CCR-5F_F2
CCR-5G_F2
CCR-5H_F2
CCR-6A_F2
PAM
CCR-F3_Wild
CCR-7D_F3
CCR-7A_F3
CCR-6H_F3
CCR-6F_F3
CCR-5C_F3
CCR-4E_F3
CCR-4B_F3
CCR-6A_F3
CCR-5H_F3
CCR-5G_F3
PAM

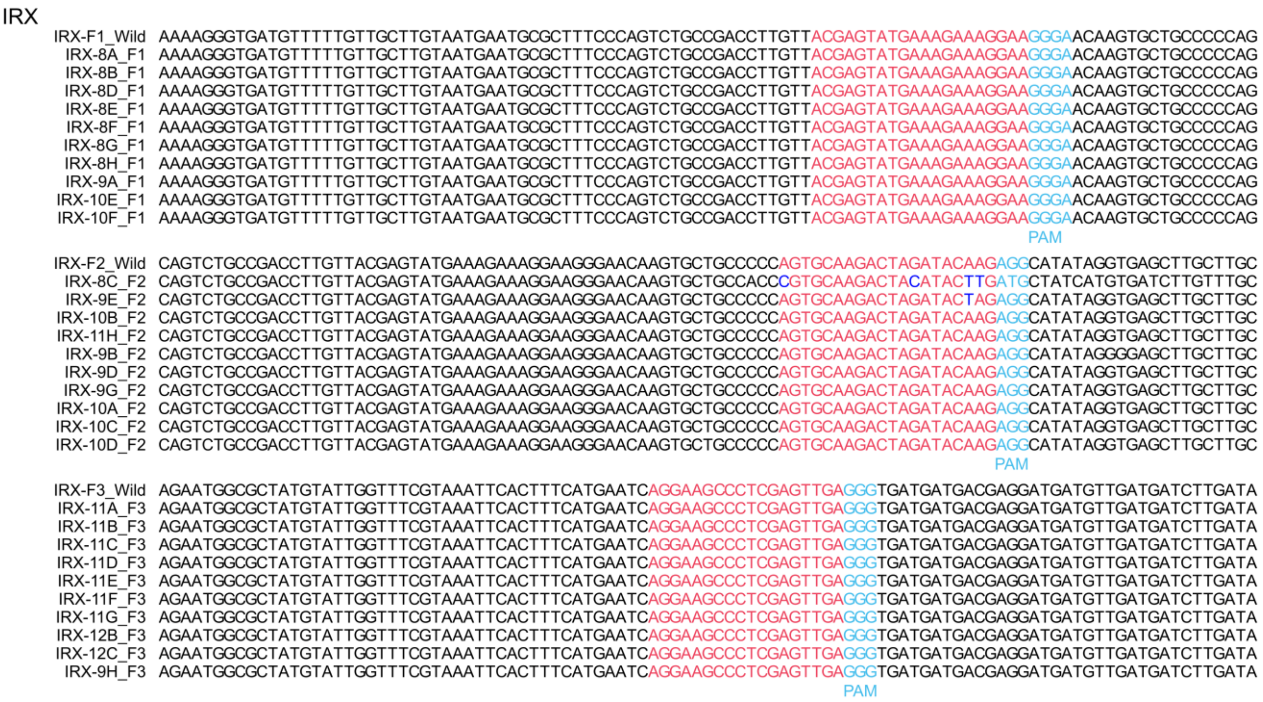

Figure 2. Target genome editing on five genes involved in lignocellulose biosynthesis pathway in *Dendrobium officinale* plants. Three target sites were selected for each gene to perform gene knockout and ten plants were sequenced for each target site. Red shadow marks the target sequence recognized by sgRNA. PAM, the protospacer adjacent motif. DNA insertions, deletions and point mutations are shown in blue color letters.

Supplemental Table: The names and components of the media.

Component	Z1	B3-3	G	B3-TS	SH4-TS	FD5-TS	SB2-TS
**Basic medium**	MS	B5	B5	B5	SH	1/2MS	B5
**Potato (g/L)**	80	40	40	40	80	80	50
**Banana (g/L)**	-	-	-	-	-	-	50

**Sucrose(g/L)**	30	25	25	25	25	25	-
**Agar (g/L)**	-	6.8	6.8	6.8	6.8	6.8	-
**Active carbon (g/L)**	-	1	1	-	-	1	2
**6-BA (mg/L)**	0.5	0.5	0.5	0.5	0.2	0.2	-
**NAA (g/L)**	0.2	0.2	0.2	0.2	0.5	0.2	0.1
**AS (umol/L)**	-	-	100	-	-	-	-
**Cef (mg/L)**	-	-	-	250	250	250	200
**TMT (mg/L)**	-	-	-	250	250	250	-
**Hyg (g/L)**	-	-	-	18	18	9	-
**Hyponex I (g/L)**	-	-	-	-	1	1	1
**KT (mg/L)**	-	-	-	-	-	-	0.1
**CH (mg/L)**	-	-	-	-	-	-	0.1
**IBA (mg/L)**	-	-	-	-	0.2	-	0.5
**PH**	5.8	5.8	5.8	5.8	5.8	5.8	5.8

6-BA: 6-benzylaminopurine, NAA: 1-Naphthaleneacetic acid, AS: acetosyringone, Cef: Cefradine, TMT: timentin, Hyg: hygromycin B, IBA: Indole-3-Butytric acid, KT: Kinetin, CH: CaseinacidHydrolysate. The tuber of potato and the pulp of banana were extracted and then juiced. Each 100 gram tissue were diluted to solution by adding 100ml water.
